# Alcohol and the Eye

**DOI:** 10.18502/jovr.v16i2.9089

**Published:** 2021-04-29

**Authors:** Saeed Karimi, Amir Arabi, Toktam Shahraki

**Affiliations:** ^1^Ophthalmic Research Center, Research Institute for Ophthalmology and Vision Science, Shahid Beheshti University of Medical Sciences, Tehran, Iran; ^2^Department of Ophthalmology, Torfeh Hospital, Shahid Beheshti University of Medical Sciences, Tehran, Iran

**Keywords:** Alcohol, Cornea, Dry Eye, Ethanol, Ethyl Alcohol, Eye, Fetal Alcohol, Glaucoma, Macular Degeneration, Methanol, Optic Neuropathy, Retinopathy, Teratogenicity

## Abstract

In this article, we present a review of ocular conditions related to alcohol consumption. A search of the literature published from 1952 to March 2020 was performed. The titles and abstracts were screened and the eligible studies were selected. PubMed, ISI Web of Knowledge database, Scopus, Embase, and the Cochrane Library were searched. We categorized the relationship between alcohol intake and ocular conditions by the type of ocular exposure to alcohol. Accordingly, ocular findings following acute alcohol intoxication, optic neuropathy following methanol toxicity, congenital conditions related to maternal alcohol consumption, and ocular disease related to chronic alcoholism are discussed. The main feature of alcohol intoxication in the eye is abnormal eye movement. Acute optic neuropathy secondary to methyl alcohol consumption is a serious ocular disease with permanent vision loss or scotoma. Prenatal exposure to ethanol may end in fetal alcohol spectrum disease, where ocular findings are a constant component. The association between chronic alcohol consumption and increased risks of cataract, age-related macular degeneration, diabetic retinopathy, different types of optic neuropathy, impairment of visual quality, retinal vascular disease, and ocular surface disease has also been reported. Along with detrimental medical and social effects, the role of alcohol consumption in different ocular conditions should be considered, as alcohol-induced visual disturbances may contribute to the heavy burden of alcohol abuse on the healthcare system and overall quality of life.

##  INTRODUCTION

As a potentially modifiable risk factor for many disorders, the importance of alcohol consumption has already been documented in a variety of medical fields. The question is whether alcohol consumption should be considered as a contributing factor of ocular diseases. Through a comprehensive review of the current literature, this article will discuss on some ocular conditions which have been reported to be associated with alcohol consumption. We have categorized the topic as follows:

i. Alcohol intoxication and the eye

ii. Acute methyl alcohol optic neuropathy

iii. Alcohol and congenital ocular diseases

iv. Chronic alcoholism and the eye

Alcohol consumption refers to the intake of drinks containing ethyl alcohol. According to the definitions in the United States, approximately 14 g of pure alcohol in any type of drink is considered as a standard alcoholic drink.^[1]^ The containing beverage can be regular beer, malt liquor, table wine, or distilled spirits.

Various studies have classified alcohol consumption differently. Alcohol consumption may be called heavy or light drinking, based on the overall measurement of the history of alcohol consumption, or may be estimated based on the number of drinks consumed per day or week [Table 1].^[2,3]^ The definition of heavy drinking varies in the literature. In this review, the concept of heavy drinking will be clarified through both quantitative and qualitative descriptions of daily alcohol consumption based on moderate or heavy alcohol abuse.

**Table 1:** Types of alcohol consumptions according to “drinking patterns and their definitions alcohol research: current reviews”^[1]^


**Type of alcohol consumption**	Definition
Low-risk drinking and alcohol use disorder	No more than 3–4 drinks on any single day and no more than 7–14 drinks per week
Moderate alcohol consumption	Up to 1–2 drinks per day
Binge drinking	4–5 drinks in a 2-hr time frame
Heavy drinking	Binge drinking on each of 5 or more days in the past 30 days

##  METHOD

Papers published from January 1952 to March 2020 was reviewed by searching ISI Web of Knowledge database, PubMed, Scopus, Embase, and the Cochrane Library. The following keywords were used: “alcohol consumption”, “ethanol”, “ethyl alcohol”, “alcohol and the eye”, “alcohol and cataract”, “alcohol and glaucoma”, “alcohol and diabetic retinopathy”, “alcohol and retinal disease”, “alcohol and dry eye”, and “alcohol and age-related macular degeneration”. No language limitation was applied. Articles and meta-analyses that published information about oral alcohol consumption and ocular disease were selected through review of abstracts, references, and titles. A total of 106 studies were included. The first study was published in 1952, and the last in 2019.

##  ALCOHOL INTOXICATION

Intoxication means the effect of acute consumption of alcohol on different physiologic processes in the body. Not only following binge drinking, it can even happen following acute intake of light or moderate levels of alcohol.^[4]^ The main feature of alcohol intoxication refers to its effect on the central nervous system. However, change in visual functions have always been focused in ethanol intoxications. Ocular findings may be related to reduction of Gamma-aminobutyric acid (GABA) activity, which is a major inhibitory neurotransmitter in the brain.^[5]^ GABA has been found in different parts of the visual pathway, from retinal ganglion and bipolar cells to the lateral geniculate nucleus, superior colliculus, and the visual cortex.^[6]^ Visual disturbance secondary to alcohol intoxication may manifest by impaired color perception,^[4]^ decreased contrast sensitivity,^[7]^ or abnormal eye movements.^[8,9,10]^ Impairment of cognitive processing in CNS may cause subclinical alterations in the eye movements. These alterations may include a high latency for fixation, increased duration of fixations, and increased frequency of saccades.^[11]^ In a study on alcohol intoxication and its ocular findings, volunteers of alcohol and placebo condition were evaluated by eye tracker to record eye movements during completion of Visual Maze Test.^[11]^ Significant differences were reported in the first fixation latency, total task time, and number and duration of fixations and saccades.

##  ACUTE METHYL ALCOHOL INTOXICATION

Methanol is a toxic alcohol found in industrial agents. Methanol toxicity can occur via inhalation, ingestion, or transdermal absorption. It is absorbed via the gastrointestinal tract in less than 10 min.^[12]^ Methyl alcohol is metabolized to formic acid, which is known to be the main mediator of permanent neurologic damage following methanol intoxication.^[13,14]^

Methanol can lead to ocular damage through two independent pathways: retinal damage and optic neuropathy. The former pathway refers to the damage to Muller cells and photoreceptors.^[15,16]^ Inner segment lesions of retinal photoreceptors have been reported to appear following methanol ingestion, where rods seems to be more sensitive to this insult, compared to cones.^[17]^ However, methanol-induced acute optic neuropathy has been studied more widely, and is considered as the main ocular damage following methanol intoxication. Ocular symptoms may occur in half of the intoxicated patients, and they often develop after 6 hr following methanol ingestion.^[18]^ It is manifested by blurry vision, visual hallucination, dense central scotoma, and decreased visual acuity. The main findings on examination include nystagmus, sluggish pupils, disc swelling, and optic disc hyperemia.^[12,17,19]^ Demyelination of the retrobulbar portion of the optic nerve is a histopathologic hallmark of the condition.^[20]^ Formic acid is believed to damage oxidative pathways through mitochondrial cytochrome C oxidase inhibition, which primarily affect susceptible zones of circulation including central nervous system and optic nerve watershed areas.^[21,22]^ In addition, interrupted axoplasmic conduction secondary to sodium–potassium adenosine triphosphatase inhibition causes axonal swelling in the optic nerve.^[20]^ Finally, increased production of reactive oxygen mediators results in neuronal lysis.^[4,14]^

Methyl alcohol intoxication is a life-threatening condition, where its acute optic neuropathy is of secondary concern. However, presence of ocular signs and symptoms may help to make an early diagnosis, thus providing opportunity for more timely therapeutic measures. Correction of metabolic acidosis, elimination of methanol from the blood using hemodialysis, and competitive inhibition of alcohol dehydrogenase by ethanol or fomepizole are the mainstays of treatment.^[13]^

Among the medications used for methanol-induced acute optic neuropathy, intravenous high-dose methylprednisolone may be beneficial in the visual recovery of these patients [Table 2]. Recently, intravenous erythropoietin (EPO) added to high-dose intravenous steroids have been postulated to be an effective combination therapy.^[23]^ In a study on the topic, 11 participants received intravenous EPO (10,000 IU twice a day) for three days as an adjuvant to methylprednisolone, while other 11 participants received methylprednisolone only. Both final BCVA and thickness of retinal nerve fiber layer were reported to be significantly better in the EPO group.^[23]^ In a prospective, non-comparative case series on 32 eyes of 16 patients,^[24]^ one or two courses of intravenous EPO (20,000 units/day for three days) appeared to improve visual acuity in patients with methanol optic neuropathy. The Erythropoietin in Methanol Associated Optic Neuropathy (EPO-MAON) trial (ClinicalTrials.gov Identifier: NCT02376881) is designed as a randomized, controlled trial to assess the efficacy of 20,000 IU EPO IV infusion for three successive days in improvement of visual outcome after three months following the treatment.

**Table 2:** A summary of studies performed on the efficacy of intravenous erythropoietin and high-dose corticosteroid as a medical treatment for methanol toxicity.


**Study**	**Year**	**Type of study**	**Number of patients**	**Treatment protocol**	**The main outcome**
Pakdel et al^[24]^	2018	Non-comparative case series	16	Intravenous recombinant human EPO consisted of 20,000 units/day for three successive days	BCVA significantly increased in the last follow-up examination
Pakravan et al^[23]^	2016	Comparative case series	11	Intravenous recombinant human EPO consisted of 10,000 IU twice a day for three days as an adjuvant to methylprednisolone	The final BCVA was significantly better in the EPO group
Yunard et al^[25]^	2016	Case series	19	Intravenous high-dose methylprednisolone with or without hemodialysis	73% of the cases showed VA improvement
Sharma et al^[26]^	2012	Case series	4	500 mg methylprednisolone twice a day for 3 days; followed by oral prednisolone 1 mg/kg/day for 11 days	100% of the cases showed VA improvement
Abrishami et al^[27]^	2011	Case series	6	250 mg intravenous methyl prednisolone every 6 hr for 4 days; followed by oral prednisolone 1 mg/kg/day for 10 days	100% of the cases showed VA improvement
Triningrat et al^[28]^	2010	Retrospective descriptive study	16	Hemodialysis and intravenous methylprednisolone 1000 mg/day for three days, followed by oral prednisone 1 mg/kg/day for 11 days	100% of the cases showed VA improvement
Shukla et al^[29]^	2006	Case series	17	Intravenous methylprednisolone 1000 mg/day for three days	82% of the cases showed VA improvement
BCVA, best corrected visual acuity; EPO, erythropoietin; VA, visual acuity					

##  FETAL ALCOHOL SYNDROME AND THE EYE

As markers of teratogenesis, presence of ocular findings are considered as a useful adjunct to the diagnosis of fetal alcohol syndrome (FAS). It is believed that eye diseases occur in over 90% of children with the FAS.^[30]^ Recently, it has been postulated that patients with fully developed FAS present the highest rate of ocular findings.^[31]^

Alcohol has been shown to cause ocular defects by affecting expression of some transcription factors such as Pax6 and Otx2,^[32,33,34]^ retinoic acid signaling,^[35,36,37]^ and reactive agents of oxygen and nitrogen signaling.^[38,39]^ Furthermore, Hug et al reported abnormal retinal function according to abnormal electroretinograms in 10 FAS patients.^[40]^ Short palpebral fissures, epicanthus, ocular hypertelorism, coloboma, strabismus, blepharoptosis, microphthalmia, and abnormalities of the fundus are ocular manifestations of FAS.^[41]^ Also, some case reports of lens opacity associated with FAS have been published.^[42]^ The most frequent findings of the posterior segment are optic disc hypoplasia and tortuosity of the retinal vasculature.^[43,44]^ In the Swedish cohort of children with FAS, the prevalence of optic nerve hypoplasia and vascular tortuosity was reported to be 48% and 49%, respectively.^[45]^

The high prevalence of ocular involvement in FAS has led the researchers to present a diagnostic tool called “4-Digit Eye Diagnostic Code.” This tool uses four main ocular manifestation of FAS, including visual acuity, refraction, strabismus, and structural abnormalities, to present a diagnostic help and guideline for follow-up examinations in FAS patients.^[31]^ Not only for diagnosis, ocular examination permits appropriate management of ocular disease in FAS, which prevents visual loss.^[41]^

More recently, modulation of ion channel has been studied for their potential ability in reducing ocular defects in FAS.^[46]^ In animal models, blue light-mediated hyperpolarization of membranes through ChR2D156A channels rescued ethanol-induced eye structural defects.^[46]^

##  CHRONIC ALCOHOLISM AND THE EYE 

### Age-related Macular Degeneration (AMD)

AMD is a frequent cause of blindness among the elderly in developed countries.^[47,48]^ Numerous epidemiological studies have supported the probable link between alcohol consumption and AMD,^[2]^ however, the pathophysiological mechanisms remain unclear. Alcohol is considered as a neurotoxin that causes brain damage via oxidative pathways. Hence, the retina might be similarly affected.^[49,50]^ Additionally, increased oxidant stress associated with excess alcohol consumption has caused tissue damage in different organs of animal models.^[51,52]^ Furthermore, due to poor nutrition in heavy drinkers, decreased intake levels of carotene and antioxidants are more likely to occur.^[53,54]^ Some protective nutrients against AMD, such as zinc and vitamin, are also lower in heavy drinkers compared to non-drinkers.^[55,56]^ Finally, alcohol has been related to formation of new vessels and progression of choroidal neovascularization in animal studies.^[57]^

A meta-analysis indicated that consumption of ≥30 g/day of alcohol was correlated to a higher risk of early AMD by 47–67%.^[58]^ Additionally, some evidence showed that the type of alcohol may be relevant. In the Beaver Dam Eye Study (BDES), drinking of neither wine nor liquor was associated with early or late age-related maculopathy, while beer drinking was related to retinal drusen in men.^[59,60]^ In the Melbourne Collaborative Cohort Study, the largest and most recent prospective study on 20,963 participants since 2003 to 2007, consuming more than 20 g of alcohol per day increased the incidence of early AMD by approximately 20% in both sexes.^[61]^ This study also revealed that not only high levels but also social or moderate levels of alcohol consumption increase the risk of early AMD.^[61]^

### Diabetic Retinopathy (DR)

Results of studies on the relationship between alcohol consumption and incidence of diabetic retinopathy (DR) have been contradictory. Literature reports that the associations are limited and findings are confusing. Among the initial studies in this field, a prospective study revealed that patients with diabetes who consumed more than 31 g of alcohol per day had a higher risk of developing DR compared to the non- to light drinkers.^[62]^ In the following studies, no correlation between baseline alcohol intake and the progression of DR was evident over more than four years.^[63,64]^ In a recent meta-analysis of 15 observational studies, alcohol intake was not associated with an increased risk of DR.^[65]^ In the subgroup analysis, no significant correlations were found between different stages of DR and various alcohol consumption groups. The main limitation of this meta-analysis was lower number of cohort studies included.

### Retinal Vein Occlusion (RVO)

Hyperviscosity and severe dehydration resulting from a high alcohol intake has been linked to central retinal vein occlusion (RVO) in young patients.^[66]^

In the Eye Disease Case–Control Study, moderate alcohol consumption was reported to be protective, as it was linked to lower odds of RVO.^[67]^ However, other studies, such as the BDES, revealed no such association between alcohol consumption and RVO.^[68]^

### Other Retinal Conditions

Alcohol is a known risk factor for central serous chorioretinopathy (CSCR).^[69]^ It may contribute to nitric oxide-related abnormalities of autoregulation of the choroidal blood vessels.^[70]^ Altered regulation of the choroidal blood flow can increase the permeability of the vasculature as well as accumulation and leakage of the fluid. Moreover, in a report of alcoholic liver disease and bilateral multifocal CSCR, the authors postulated that end-stage liver disease secondary to alcoholism could be the etiology of CSCR.^[71]^

In BDES, drinking more than four drinks/day of alcohol was associated with asteroid hyalosis.^[72]^

Adolescence is a period of important neurological development, and alcohol exposure in this period may cause some visual defects, particularly defects of color vision, which is more sensitive to neurotoxicants.^[4]^ The visual scotopic performance also worsens with heavy alcohol consumption.^[73]^ In a study on the topic, both the retinal image quality and visual performance under low-scotopic conditions were worsened after acute alcohol consumption. Increased pupil size and tear film disturbances were presented as the etiological factors of decreased optical quality.^[73]^

### Alcohol-related Optic Neuropathy

Toxic/nutritional optic neuropathy secondary to chronic alcohol consumption is characterized by central or cecocentral scotoma due to papillomacular bundle damage, associated with color vision defects.^[74]^ A detailed history and ocular examination, color vision and visual field tests, and laboratory investigations for serum B12 and folate levels are required to obtain the diagnosis.^[18]^ The management includes reduction or cessation of alcohol intake, and vitamin B12 and folate supplementation.^[75]^

### Glaucoma

Results of the Framingham Eye Study were conflicting, as they suggested that high alcohol consumption might be associated with glaucoma.^[76]^ In the Long Island Glaucoma Case–Control Study,^[77]^ alcohol consumption was more frequent in patients with higher intraocular pressure (IOP) compared to the controls. In a hospital-based study in Japan, men with alcohol consumption showed a high risk of elevated IOP.^[78]^ In this study, 569 men and women participated, where alcohol consumption had a significantly positive association with the IOP in men. Interestingly, women did not show this association. A similar sex-related difference was reported in the Barbados Eye Study: current alcohol consumption was positively related to IOP in men. Notably, the Barbados Study included black patients without glaucoma, and as a superiority over Framingham Study, the results were reported after controlling for obesity and hypertension.^[79]^ Similar results were reported in the studies of the Chinese population: Chinese men >65 years who were current alcohol consumers had higher IOP compared to female consumers.^[80]^ It is notable that in the latter study, non-contact tonometry was used to measure the IOP.

On the other hand, acute alcohol ingestion reportedly lowers IOP in both healthy and glaucomatous eyes.^[81,82]^ Both hyperosmotic effect and suppression of vasopressin by ethanol in the blood reduces blood flow and net water transport into the eye.^[83]^ Moreover, an inhibitory effect on the secretory cells in the ciliary process is reportedly related to alcohol intake.^[84]^ Although alcohol may decrease the blood flow of the anteriorly located ciliary body, posterior structures, such as the optic nerve, may receive more blood when alcohol is present in the circulation. This mechanism can be protective against the development of primary open-angle glaucoma (POAG).^[85]^ Along with these basic evidences, a subset of case–control studies reported the protective effects of alcohol consumption against the incidence of POAG and elevated IOP.^[86,87]^ In a study on 100 age- and sex-matched ocular hypertensive patients and 100 ocular normotensive patients, absence of liquor consumption was significantly associated with the presence of ocular hypertension.^[87]^

Between these two extremes of converse results, various epidemiological studies reported no association between alcohol and IOP elevation or glaucoma.^[88]^

The only report on the longitudinal correlation of IOP and alcohol consumption was delivered by the Barbados Eye Study,^[89]^ in which alcohol consumption at baseline was not associated with a higher four-year risk of elevated IOP.^[89]^ Additionally, in a large prospective study on 856 participants including both sexes, chronic alcohol intake <30 g/day did not influence the risk of POAG.^[90]^ Accordingly, the relationship between alcohol consumption and the risk of glaucoma requires a more prospective investigation with detailed assessments on alcohol use.

### Alcohol and Cataract

A recent systematic review and meta-analysis revealed that heavy alcohol consumption significantly increased the risk of age-related cataract.^[91]^ In this meta-analysis which was performed on 10 studies, the correlation of moderate alcohol consumption and senile cataract were marginally non-significant. Heavy alcohol consumption could produce free radicals in the liver. Transmission of the products to the lens may lead to aggregation of lens proteins, leading to cataract formation.^[91,92]^ Acute alcohol exposure increases intracellular lens calcium levels through inhibition of the calcium pumps on lens fibers.^[93]^ This disturbance in calcium homeostasis may lead to cataract formation.

**Table 3:** Some ocular conditions related to alcohol consumption


**Type of alcohol consumption**	**Ocular findings**
Acute alcohol intoxication	Abnormal eye movements, altered color perception, decreased contrast sensitivity
Acute methanol optic neuropathy	Optic disc edema, retinal ganglion cell damages, permanent scotoma or vision loss
Alcohol teratogenicity	Short palpebral fissures, epicanthus, ocular hypertelorism, coloboma, strabismus, blepharoptosis, cataract, microphthalmia, hypoplasia of the optic nerve, tortuosity of the retinal vessels
Chronic alcoholism	Anterior segment Cataract Dry eye syndrome Corneal epitheliopathy Intraocular pressure Primary open angle glaucoma Optic nerve Alcohol-induced optic neuropathy Retina and choroid Age-related macular degeneration Diabetic retinopathy Retinal vein occlusion Central serous chorioretinopathy Functional retinal disease Asteroid hyalosis

### Alcohol and ocular surface disease

Although topical administration of ethanol is a known cause of keratopathy and has been used for epithelial cell removal in different ocular surgeries,^[94,95]^ controversial opinions on the association between oral alcohol and ocular surface disease have been published. In a case–control study, 0.75g/kg of oral ethanol was administered to 10 volunteer men at 8 pm.^[96]^ Presence of ethanol in tears, tear osmolarity, Schirmer test, and tear break-up time (TBUT) were evaluated at midnight and the next morning. Ethanol was detected in tears at midnight, while it was undetectable in the tear samples collected next morning. The authors reported that the presence of alcohol in tears was associated with increased tear osmolarity, decreased TBUT, and staining of ocular surface with fluorescein. Schirmer test and corneal sensitivity showed no change. In other publications, Chia et al^[97]^ argued the protective role of alcohol in the development of dry eye syndrome (DES), while Galor et al^[98]^ showed that drinking increases the risk of DES. The BDES reported a higher rate of DES symptoms in individuals with a history of heavy alcohol consumption.^[99]^ A comparative case–control study on men with alcohol consumption more than four drinks/day reported decreased Schirmer test result and TBUT, in addition to changed impression cytology of the conjunctiva.^[100]^ Some other researchers believed that DES may have no association with alcohol consumption.^[101,102,103]^

The results of a recent meta-analysis involving nine cross-sectional and one case–control studies indicated that alcohol consumption could increase the risk of DES, irrespective of age and sex.^[104]^ Tear hyperosmolarity following alcohol consumption has been detected after heavy drinking.^[73,105]^ Moreover, ethanol induces the expression of interleukin-1, interleukin-6, and interleukin-8 in the ocular surface cells.^[106]^ As an additional mechanism, chronic alcoholism can induce vitamin A deficiency by reducing retinol in the liver. Absence of vitamin A causes increased epidermal keratinization through loss of the goblet cells from the cornea and conjunctiva, which is a major pathogenesis of DES.^[107,108]^

##  DISCUSSION

Many pathophysiological aspects of the deteriorating effects of alcohol consumption on ocular structures have been demonstrated, however, additional well-designed studies are required for a definite conclusion. According to the present medical literature, along with harmful effects on many other organs of the body, alcohol consumption may damage ocular tissues, from the cornea and conjunctiva to the retina and optic nerve. Among them, alcohol teratogenicity and alcohol-related optic neuropathies are more definite and seem to be the most serious ocular conditions related to alcohol consumption [Table 3].

Ophthalmologists should consider alcohol consumption as a modifiable risk factor for ocular disease.

Future direction on clinical aspects of the topic may be as follows:

i. How to reduce the adverse effects of prenatal alcohol exposure in pregnant alcohol drinkers?

ii. How to optimize the management of acute methanol toxicity and its optic neuropathy to preserve a higher level of vision and visual function?

iii. How oral ethanol can expose heavy drinkers to ocular surface disease?
